# Optimal Design of Sustainable Reinforced Concrete Precast Hinged Frames

**DOI:** 10.3390/ma16010204

**Published:** 2022-12-26

**Authors:** Andrés Ruiz-Vélez, Julián Alcalá, Víctor Yepes

**Affiliations:** Institute of Concrete Science and Technology (ICITECH), Universitat Politècnica de València, 46022 Valencia, Spain

**Keywords:** reinforced concrete, precast, hinged frame, metaheuristic, optimization, sustainability

## Abstract

Sustainable development requires improvements in the use of natural resources. The main objective of the present study was to optimize the use of materials in the construction of reinforced concrete precast hinged frames. Proprietary software was developed in the Python programming language. This allowed the structure’s calculation, verification and optimization through the application of metaheuristic techniques. The final cost is a direct representation of the use of materials. Thus, three algorithms were applied to solve the economic optimization of the frame. By applying simulated annealing, threshold accepting and old bachelor’s acceptance algorithms, sustainable, non-traditional designs were achieved. These make optimal use of natural resources while maintaining a highly restricted final cost. In order to evaluate the environmental impact improvement, the carbon-dioxide-associated emissions were studied and compared with a reference cast-in-place reinforced concrete frame. The results showed designs with reduced upper slab and lateral wall depth and dense passive reinforcement. These were able to reduce up to 24% of the final cost of the structure as well as over 30% of the associated emissions.

## 1. Introduction

The continuous improvement and widespread dissemination of knowledge about environmental issues raise awareness in a global population that seeks more sustainable development [1,2]. The growth and evolution of societies are directly related to their infrastructure. Thus, the construction, maintenance and demolition of the structures that support a country’s economic and social practices are activities intrinsic to its development [3]. The characteristic consumption of large quantities of materials places construction as one of the industries that produce more greenhouse gas emissions, generating around 5% of the total CO_2_ emitted yearly [4]. Concrete is currently the most widely used material in construction. Therefore, a significant portion of the emissions is derived from its use [5,6,7].

In this context, it is feasible to affirm that construction is an activity inherent to human development. However, the intrinsic need for these related activities should not justify an inefficient use of natural resources. Hence, in the last decades, there has been a clear trend towards reducing the use and improving the characteristics of the materials necessary for constructing the structures surrounding us [8,9,10]. Using sustainable materials in conjunction with recycled materials allows direct reductions in the associated CO_2_ emissions [11,12,13]. However, the real-world application of these ways of improvement is limited by the need to develop comprehensive experimental studies that establish conditions of use and appropriate practical recommendations [14]. Thus, the experimental nature of these studies means that the implementation period of these solutions is considerably affected.

Considering the aforementioned limitations, several studies have focused on optimizing the structural design process [15,16,17,18]. An improvement in the design can lead to immediate improvements in the social and environmental impact associated with its construction [19]. Traditionally, the design process has been very much linked to the engineer’s theoretical knowledge, technical skills and previous experience [20]. This methodology starts with the complete definition of the different structural sections, which comprehends materials selection and the establishment of passive reinforcement configurations. Once defined, compliance with the limit states stipulated in the applicable code is verified. If the current structure does not comply, the type or quantity of materials is modified for further verification. This process is repeated on a trial-and-error basis until a final design is selected. Although this practice allows for achieving structurally safe designs, it does not comprehend the consideration of optimal material usage.

Interest in designing sustainable structures, together with the improvement of the computational tools currently available, has led to the development of a series of new design methodologies. Consisting of the application of metaheuristic techniques in the design of structures, these methodologies result in new designs. These, in addition to complying with the relevant limit states, manage to minimize or maximize a series of previously established factors such as economic cost, associated CO_2_ emissions or the energy consumed in the construction of the designed structure [3]. These techniques have been highly improved during the last decade, mainly due to the simplicity of the algorithms and their high adaptability, in addition to the ability to avoid convergence to a local optimum. Due to the importance of improving the design process and the positive characteristics of the aforementioned methodologies, several studies have applied heuristic and metaheuristic optimization to design concrete structures, such as prestressed bridges [21], retaining walls [20,22,23], bridge piers [24] and building structures [25,26]. The results establish a direct relationship between the final cost of the structure and the CO_2_ emissions associated with its construction. The optimization of precast girder bridges carried out by Yepes et al. [27] concludes that a reduction of EUR 1 in the final cost of the bridge equals the avoidance of emitting 1.74 kg of CO_2_.

**Figure 1:**
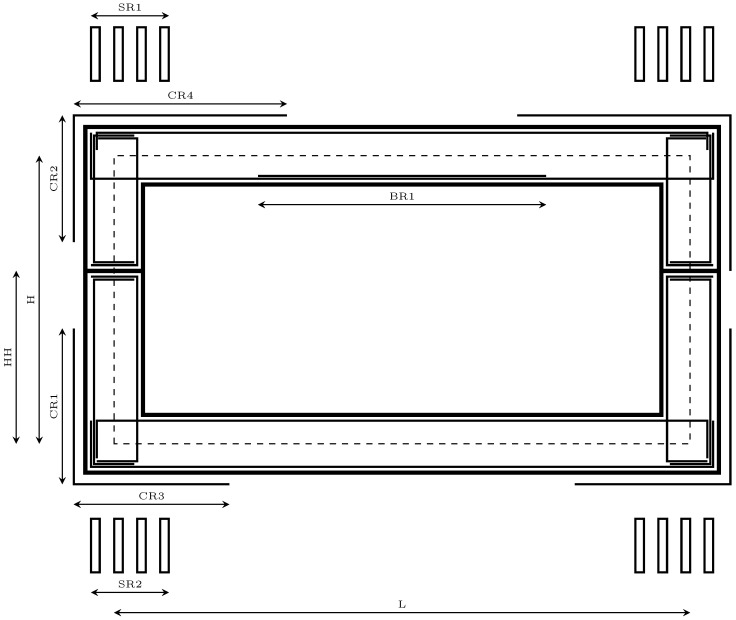
Parameters of the RCPHF considered in the optimization problem.

The use of the reinforced concrete precast hinged frames (RCPHF), considered in the present study, is widely extended in transportation infrastructures. Mainly applied as a solution for the crossing of traffic lanes with the main road, these prefabricated and reinforced concrete cast-in-place frames (RCCPF) typically cover spans between 3 and 20 m [28]. The RCPHF is a specific type of bridge structure that is particularly suitable when there is a low-bearing strength terrain or when the location happens to be a flood zone, something that could lead to a higher scour risk. The structure comprises three distinct parts, the top slab, the lateral walls and the bottom slab. The traditional design establishes the top and bottom slab depth between 1/10 and 1/15 of the span. In the case of the sidewalls, depth is established between 1/12 of the span and the depth considered for the slabs. In practice, the precast structural assembly is separated into two sets, allowing its transport from the prefabrication plant to its final site. Thus, the design of this structural typology is mainly determined by four factors: the horizontal span (*L*), the required height (*H*), the earth cover (*HE*), and the height of the hinge (*HH*). Figure 1 shows a graphic representation of the structural typology considered in this context.

The design optimization of RCCPF with similar characteristics obtains high-quality results [29,30]. However, there happens to be a lack of further development in the study of RCPHF applying current methodologies. The modular nature of precast structures, in conjunction with the greater quality and continuity of the prefabricated products when compared to cast-in-place structures, supports the relevance of studying the RCPHF as a better solution for transportation infrastructures [31,32,33]. In this context, the present study aims to optimize materials used to fabricate the RCPHF by applying three metaheuristic algorithms. In addition, a comparison between the optimized RCPHF and a reference RCCPF is carried out. The applied methodology consists of the initial development of proprietary software for stress calculation, structural and limits compliance verification and subsequent optimization through the application of heuristic algorithms. Once developed, the software is used to study the economic optimization of the RCPHF by applying simulated annealing, threshold accepting and old bachelor’s acceptance algorithms. By doing so, the present study solves the lack of existing development, bettering the current knowledge and obtaining a series of practical recommendations applicable to the design of RCPHF. By comparing the precast and cast-in-place options, the study provides a comprehensive overview of the characteristics of each typology and the environmental impact associated with each. In addition, the software developed improves the application of heuristic techniques, allowing the further study of other relevant typologies of the study field while maintaining reasonable computational costs.

## 2. Optimization Problem and Computational Model Definition

The combinatorial optimization problem developed in the present study consists of the economic optimization of RCPHF. Generally, an optimization problem aims to minimize the value of a specific objective function while satisfying a series of previously established constraints [34]. In this context, the present optimization considers the final cost of the frame, calculated according to Equation (1), as the objective function of the problem. The evaluation consists of the direct calculation of the product of each material’s unit cost $c_{i}$ multiplied by the quantity used of each of the materials $m_{i}$. In addition, based on the aforementioned need to design structures that incur lower environmental impact, the CO_2_ emissions associated with each design are evaluated by means of Equation (2). In this equation, similarly to the final cost, total CO_2_ emissions are calculated as the product of the emissions associated with each material $e_{i}$ multiplied by the quantity used of each of those materials $m_{i}$. The values considered both in the evaluation of the objective function and the calculation of the associated CO_2_ emissions are summarized in Table 1. These were obtained from the Construction Technology Institute of Catalonia by the BEDEC database [35]. The RCPHF design is subject to compliance with the requirements established by the standard regulations [36,37,38]. These, added to a series of technical considerations necessary for the complete verification of the RCPHF, are expressed in general terms through Equation (3).

(1) $$\begin{array}{c} \begin{array}{c} C\left(\vec{x}\right)=\sum_{i=1}^{n}c_{i}\cdot m_{i}\left(\vec{x}\right) \end{array} \end{array}$$

(2) $$\begin{array}{c} \begin{array}{c} E\left(\vec{x}\right)=\sum_{i=1}^{n}e_{i}\cdot m_{i}\left(\vec{x}\right) \end{array} \end{array}$$

(3) $$\begin{array}{c} \begin{array}{c} R(\vec{x})\leq 0 \end{array} \end{array}$$

Once the general principles of the considered optimization problem have been detailed, the next step is the complete definition of the problem. This consists of considering, modelling and correctly defining the RCPHF parameters, variables and constraints. The parameters are magnitudes whose values are fixed throughout the optimization process. Constituted by known information, the value of the parameters is not subject to optimization. In conjunction with the parameters, the variables allow the complete definition of the considered structure. The set formed by the different values that the variables can adopt throughout the optimization process is the so-called solution space of the problem. Finally, the set of parameters and variables that define the design must comply with a series of constraints. As stated previously, these must ensure that the optimal design represents reality, meets the structural requirements and verifies the standard limit states [36,37]. The following sections are dedicated to the description and complete definition of each component for the considered optimization problem.

### 2.1. Parameters

Starting with the parameters considered, it is relevant to reiterate that in conjunction with the variables these must provide all the information required to define the designed structure completely. Thus, the parameters considered in the present study must define the geometric characteristics of the frame, the acting actions and safety coefficients, ambient conditions and any other characteristic whose definition is necessary for the structural calculation and verification process.

There are three main geometrical parameters, consisting of the horizontal free span (*L*), the vertical free height (*H*), and the height at which the hinge is located (*HH*). In addition, following the standard process, the calculation is made per linear meter. Thus, all of the sections are one meter thick. In relation to the passive reinforcement of the structure, a series of parameters must be defined in order to establish the arrangement of the shear reinforcement (*SR*1–2), as well as the lengths of the corner reinforcement (*CR*1–4) and the negative bending reinforcement in the upper slab of the RCPHF (*BR*1). Due to the symmetrical nature of the structure, it is possible to completely define the passive reinforcement design by parameterizing one of its halves. Then, the other half is defined by considering the equivalent values. The complete set of parameters mentioned above, together with the designation established for each, is represented in Figure 1.

Additionally, it is necessary to establish various parameters related to the acting loads. One of the most determining considerations in the design of the considered structural typology is the depth of the earth cover above the RCPHF (*HE*). In this context, the first of the following parameters to define corresponds to this magnitude, and the value considered is 1.5 m. In addition, it is necessary to establish the specific weight of reinforced concrete and earth backfill, a material for which it is also necessary to define the ballast coefficient and the internal friction angle. Several parameters related to the standard regulations are also necessary. In the present study, the standard forms the regulatory basis for the acting loads, the environmental exposure directly related to the passive reinforcement cover and the load combination factor and material partial safety coefficients [36,37,38]. The global set of parameters and the values established for the optimization are presented in Table 2.

Adequately representing reality is complex. Thus, the mathematical modelling of the design process of RCPHF is an intrinsically complicated task. In this context, it is relevant to mention that during the modelling process of the problem it is necessary to make several hypotheses regarding the described parameters. First, based on the prefabricated nature, added to the consideration that sufficient expansion joints are present, it is possible to disregard the rheological effects and thermal actions. Furthermore, the magnitude of the foundation’s differential settlement is also disregarded. It is also considered that the frame’s location is a non-seismic zone, so the possible effect of the seismic actions can be neglected within the scope of the present study. Finally, common-use Spanish recommendations are considered to assess the overload associated with the Marston Effect and a heavy vehicle load [28,38].

### 2.2. Variables

**Figure 2:**
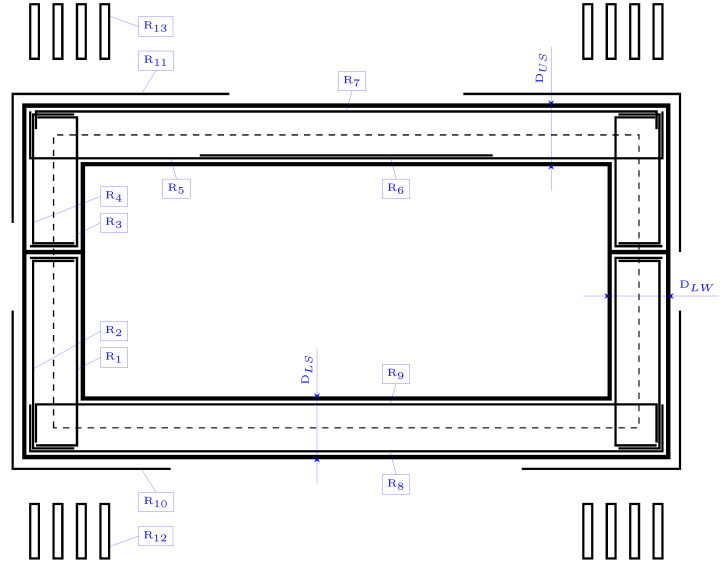
Set of variables considered in the RCPHF optimization problem.

Once the parameters are detailed, this section focuses on defining the variables. A total of 31 design variables are considered in the economic optimization of the RCPHF. These include three geometrical variables that define the depth of the upper slab ($D_{US}$), the bottom slab ($D_{LS}$) and the lateral walls ($D_{LW}$). Two variables relate to the concrete and passive reinforcement material grades, and a set composed of the remaining 26 variables relates to the passive reinforcement configuration. This set, represented in Figure 2, allows the complete definition of the passive reinforcement design corresponding to the diameter and number of bars of the longitudinal reinforcement and the diameter and branch spacing of the shear reinforcement of the structure.

It is relevant to note that each of the optimization variables is discretized. The variables corresponding to the width of the slabs and lateral walls can adopt values every two centimetres within the stipulated range. In addition, the shear reinforcement branches can take spacing values every five centimetres within the limit values. The diameter of the passive reinforcement can vary along the standard’s values between 10 and 35 mm, and the number of bars can be any integer within the defined range. In this context, the set of variables considered, together with the number of values they can adopt during the optimization process of the frame and the limit values established, are listed in Table 3.

### 2.3. Constraints

As mentioned in Section 2, the structure object of the optimization process must comply with a series of constraints represented by Equation (3). In the case of the RCPHF, the considerations to be taken into account are those established by the regulations [36,37]. In addition, the Spanish recommendations were considered [28,38]. The constraints can be broadly classified into two separate groups. The first corresponds to the Ultimate Limit States (ULS), and the second one to the Service Limit States (SLS). The ULS are constraints that must ensure the structural resistance and integrity of the frame when it is under stress incurred by the acting loads. The SLS are responsible for ensuring the correct serviceability of the structure during its service life. A step prior to the verification of the ULS and SLS compliance is the calculation of the RCPHF stress and displacements. A two-dimensional model of the frame has been considered for the linear elastic analysis of the structure.

Employing the traditional design process, the passive reinforcement is initially defined so that the flexural ULS are verified. Then, the cracking SLS compliance is checked, and the shear reinforcement is sized to verify the shear ULS without modifying the defined flexural reinforcement. This procedure leads to structural designs that comply with the standard and are structurally sound. However, these do not make optimal use of the required materials. In this context, the proposed combinatorial optimization does not contemplate the frame’s passive reinforcement design under traditional criteria. This raises considerations such as eliminating or significantly reducing shear reinforcement to be replaced by localized increases in flexural reinforcement. Such considerations can lead to non-traditional designs with reduced costs and lower environmental impact in terms of associated CO_2_ emissions, something relevant to the sustainability considerations presented in Section 1.

In the present study, the initial verification consists of the compliance of the shear ULS by comparison with the compression and tensile exhaustion limit values. This process also includes calculating the tensile stress increase in the flexural reinforcement derived from the shear stress. This increase is then considered by applying the proportional offset to the bending moment law, appropriately increasing the magnitude of the bending moment in each affected section. The next step consists of the verification of the flexural ULS. In order to do so, the calculation of the interaction diagram of each of the study sections is carried out. After doing so, it is verified that the point defined by the acting axial force and bending moment is located within this diagram. Once the flexural ULS is verified, the cracking SLS is checked by calculating the crack opening and comparing it with the maximum standard value for the environmental conditions. Next, the deformed state of the structure is checked by limiting the maximum displacement in the central section of the upper slab to 1/250 times the horizontal span. In addition, compliance with the minimum amounts of longitudinal and shear reinforcement is verified, as well as the reinforcement for cracking control. Due to the consideration of a two-dimensional analysis, which does not allow the study of the transversal stresses, the transversal reinforcement is calculated as a result of the longitudinal design, complying with the minimum standard values.

The verification process is carried out by means of the developed software, which considers a model consisting of 40 members and nodes. The stresses of the RCPHF are calculated, and then the geometric and constraints are checked. After the verification, the software provides results regarding the material measurements, final cost, associated CO_2_ emissions and a series of structural verification coefficients. These represent the relation between the stress associated with the acting loads ($A_{s}$) and the resistant limit of the structure ($R_{s}$), which corresponds to Equation (4).

(4) $$\begin{array}{c} \begin{array}{c} \frac{A_{s}}{R_{s}}\geq 1 \end{array} \end{array}$$

If all the verification coefficients are greater or equal to one, the RCPHF verifies the required ULS, SLS, minimum and maximum reinforcement amounts and the necessary geometrical checks.

### 2.4. Computational Model

The developed software obtains the deflections and internal stresses of the structure by applying the displacement method. This method consists of the solution of the matrix Equation (5).

(5) $$\begin{array}{c} \left|F\right|=\left[K\right]\cdot \left|U\right|+\left|F_{0}\right| \end{array}$$

The procedure starts with the complete definition of the structure’s stiffness matrix K. This is obtained by assembling each member’s stiffness matrices according to their position in the model, considering the member’s characteristics at both end nodes. The term $F_{0}$ represents the perfect embedding forces vector, while U corresponds to the structure’s displacements vector. The product of the stiffness matrix and the displacement vector, subsequently adding the perfect embedding forces vector, allows obtaining the structure’s internal forces vector F.

The internal stress vector is calculated for each of the RCPHF load cases. Afterwards, based on the linear elastic hypothesis, it is possible to apply the superposition principle. This way, the load stresses are combined to obtain the complete set of design stress envelopes.

## 3. Methodology

The three metaheuristic techniques applied to solve the optimization problem are simulated annealing, threshold accepting and old bachelor’s acceptance. These are algorithms with relatively simplistic operating principles that allow the resolution of complex combinatorial optimization problems. While obtaining high-quality solutions, these techniques cannot ensure convergence to the global optimum of the considered problem.

Based on the local search principle, the algorithms start from a randomly generated initial solution to which a previously established movement is applied to generate a new solution within the neighbourhood of the current one. The value of the objective function is evaluated and compared with the current one. In case of improvement, when compared to the current solution, the new solution is accepted directly. However, the interesting feature of the three techniques applied in the present study is the ability to accept solutions that worsen the current one. This is performed according to an established criterion specific to each metaheuristic algorithm. This criterion can be probabilistic, as in the case of simulated annealing, or deterministic, as in the case of threshold accepting and old bachelor’s acceptance algorithms. This way, premature convergence to low-quality optima can be avoided, allowing the algorithm to explore the solution space thoroughly.

In the present study, due to the number of design variables and the set of values that each of them can adopt during the optimization process, the solution space has a dimension of $2.1\times 10^{28}$. The magnitude of this solution space makes it feasible to state that a complete exploration does not conform to a possible way to improve the design of the considered structural typology. In this context, thanks to the aforementioned ability to converge to high-quality optimums while maintaining adequate computational costs, metaheuristic algorithms are an excellent tool for solving combinatorial optimization problems with similar characteristics [15].

### 3.1. Simulated Annealing Algorithm

Turning to the details of the algorithms applied, the first is simulated annealing, SA henceforth. First proposed by Kirkpatrick et al. [39], the SA algorithm operates based on the physical modifications at a microstructural level that the materials undergo during the thermal annealing process. In this process, the materials are subjected to a high initial temperature and then cooled slowly at a cooling coefficient rate. In the beginning, the material presents configurations with high internal energy. However, during the cooling, the microstructure undergoes a series of modifications in search of the fundamental state, a stable crystallization state with the lowest internal energy associated. Thus, during the cooling, the material passes through crystallization states with decreasing internal energies. This process is governed by the Boltzmann energetic Equation (6).

(6) $$\begin{array}{c} \begin{array}{c} e^{-(\Delta E/T)} \end{array} \end{array}$$

In this equation, ΔE is the energy increment of the new configuration, and *T* is the temperature at that time of the process. In this context, the SA algorithm resembles each of the combinatorial optimization problem’s feasible solutions with the different crystallization states of the material. Thus, the value of the objective function for each of these solutions is equated to the internal energy associated with that particular crystallization state.

**Figure 3:**
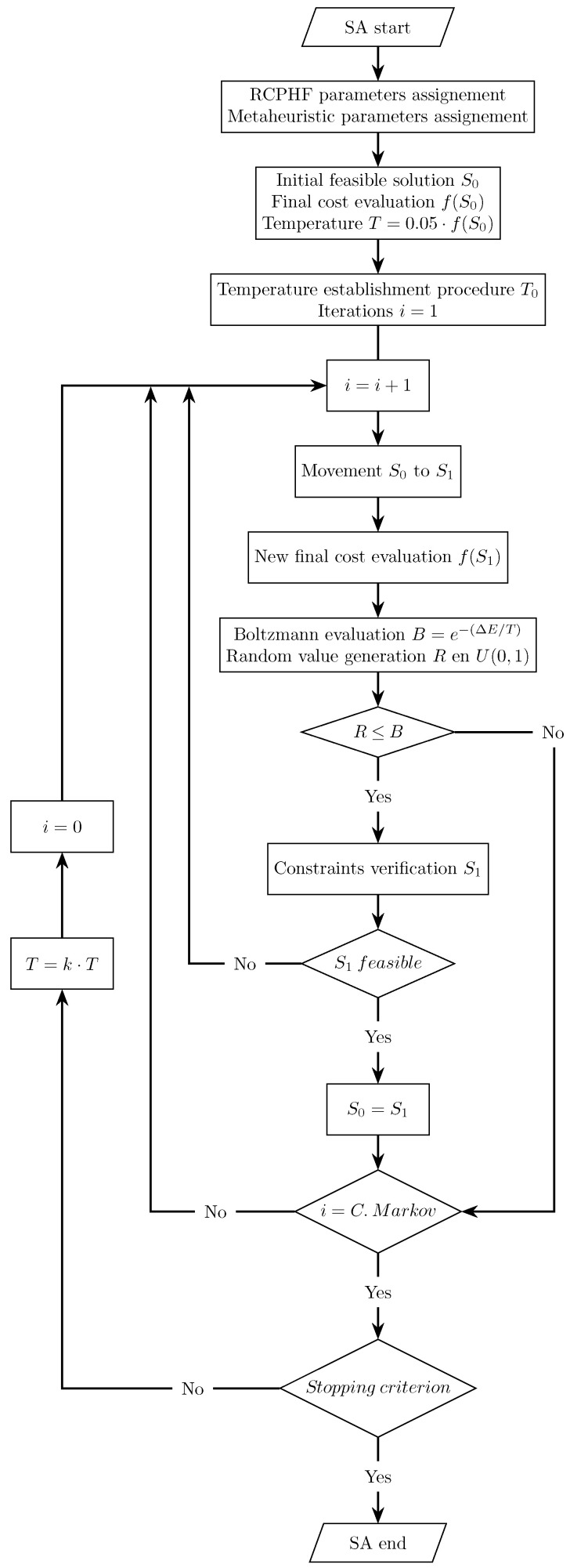
Flowchart of the simulated annealing (SA) process.

The algorithm’s operation, represented in the flowchart corresponding to Figure 3, parts from a randomly generated initial feasible solution $S_{0}$ for which the objective function, $f(S_{0})$, is evaluated. A new solution, $S_{1}$, is then generated by applying a movement, and its objective function value, $f(S_{1})$, is then evaluated. This movement consists in varying the magnitude of a certain number of the variables defining the current solution. Those solutions that present a lower cost than the current one are accepted directly. In the case of presenting a cost that involves a worsening of the current solutions, new solutions are accepted when the Boltzmann equation presents a value greater than a randomly generated value between 0 and 1. After this, the feasibility of the new solutions is evaluated by verifying compliance with the imposed constraints. If a feasible solution is found, the new solution, $S_{1}$, becomes the current solution, $S_{0}$. This process is repeated at a constant temperature for a specific number of iterations, denoted as Markov’s chain. After the completion of Markov’s chain, the temperature of the problem is geometrically reduced by applying the cooling coefficient *k*.

The algorithm’s evolution and exploration of the solution space are highly conditioned by the correct establishment of the complete set of parameters that define it. In the case of the initial temperature, the approach proposed in Medina [40] is considered since it has proved to function in a correct manner in previous studies of similar characteristics [20,23]. The optimization process finishes once the temperature of the problem is equivalent to a small percentage of the initial temperature or when, during a certain number of consecutive Markov chains, a new solution that improves the current one is not found.

The quality of the results obtained by applying the SA algorithm in the resolution of the combinatorial optimization problems of similar characteristics to the one considered in the present study directly depends on the correct calibration and definition of the metaheuristic parameters [21]. Thus, in order to achieve adequate performance, nine different metaheuristics are developed. These result from combining Markov chains between 500 and 5000 iterations and cooling coefficients between 0.80 and 0.95. Based on the results obtained in studies with similar characteristics, a maximum number of five Markov chains in which the new solutions do not improve the current ones is established as a stopping criterion. In addition, a minimum temperature equivalent to 5% of the initial temperature of the problem is set as a termination criterion.

**Figure 4:**
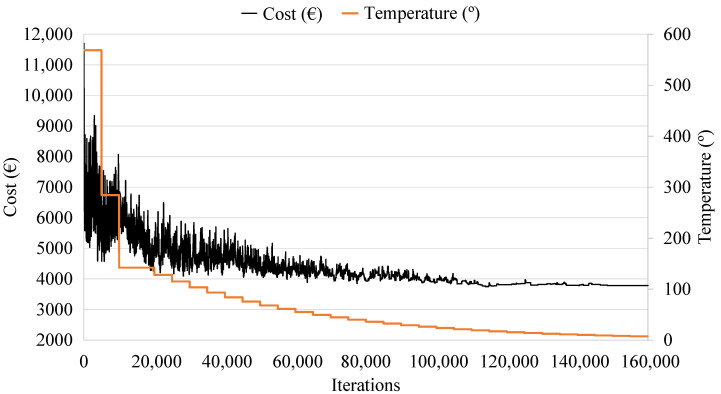
Simulated annealing trajectory for the cost and temperature as a function of iterations.

Figure 4 represents the RCPHF cost evolution for a generic execution of the developed SA algorithm. It shows the process of setting the initial temperature, the exploration of the solution space, as well as the intensification phase, where the algorithm studies the high-quality optimum towards which it has converged.

### 3.2. Threshold Accepting Algorithm

**Figure 5:**
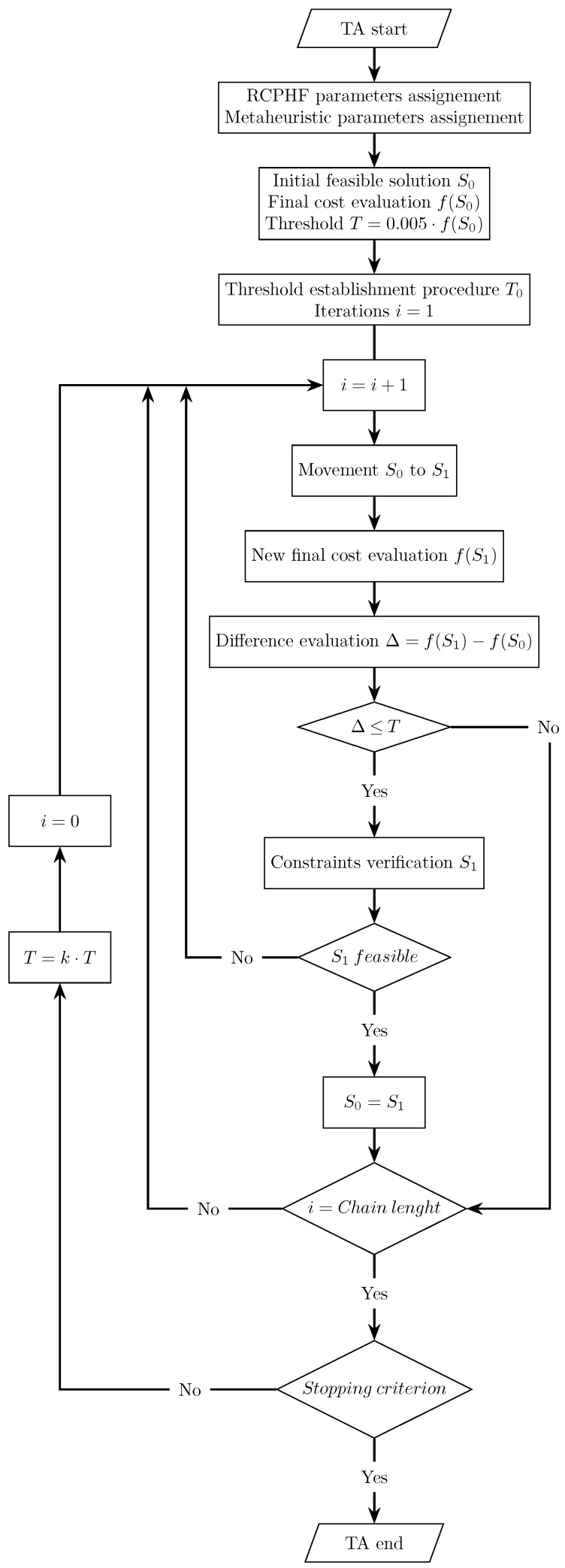
Flowchart of the threshold accepting (TA) process.

The threshold acceptance algorithm, TA henceforth, is the second of the metaheuristics applied in resolving the problem. Developed by Dueck and Scheuer [41] as an alternative to the SA, the TA presents a similar operation to the one described for SA. The TA algorithm starts with a randomly generated initial feasible solution $S_{0}$ for which the value of the objective function is evaluated. After this, a new solution is generated by applying a move, and the value of the objective function $f(S_{1})$ is evaluated. If it presents a lower cost, it is directly accepted. Otherwise, the new solution is accepted if the cost increase, when compared to the current one, is smaller than a certain threshold. The feasibility of the new solution is then evaluated and accepted as the current solution if the imposed set of constraints is verified. This process is repeated for the same threshold for a given number of iterations called chain, after which the threshold is geometrically reduced at a rate of the threshold reduction coefficient [34,42]. It can be noted that the SA and TA algorithms operation is quite similar, differing in the fact that the acceptance criterion of the SA is probabilistic, while the TA is deterministic. The detailed operation of the developed TA can be seen in the flowchart presented in Figure 5.

**Figure 6:**
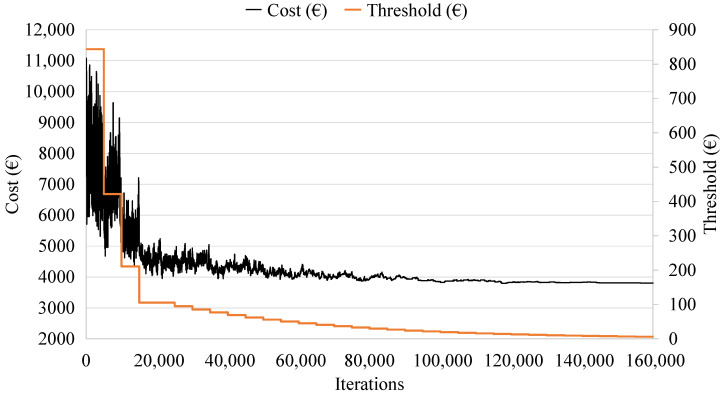
Threshold accepting trajectory for the cost and temperature as a function of iterations.

Similar to the SA algorithm, nine different metaheuristics are created, combining chain lengths between 500 and 5000 iterations and threshold reduction coefficients between 0.8 and 0.95. In addition, stop and termination criteria analogous to those detailed for the SA algorithm are established. Figure 6 shows a generic cost evolution for the programmed TA algorithm. This clearly distinguishes the initial threshold setting phase, followed by the exploration phase, where the algorithm evaluates RCHPC designs with fundamentally different characteristics thanks to the fact that its capacity to accept solutions that worsen the current one is relatively high at that time. Finally, the intensification phase is initiated towards a high-quality optimum.

### 3.3. Old Bachelor’s Acceptance Algorithm

**Figure 7:**
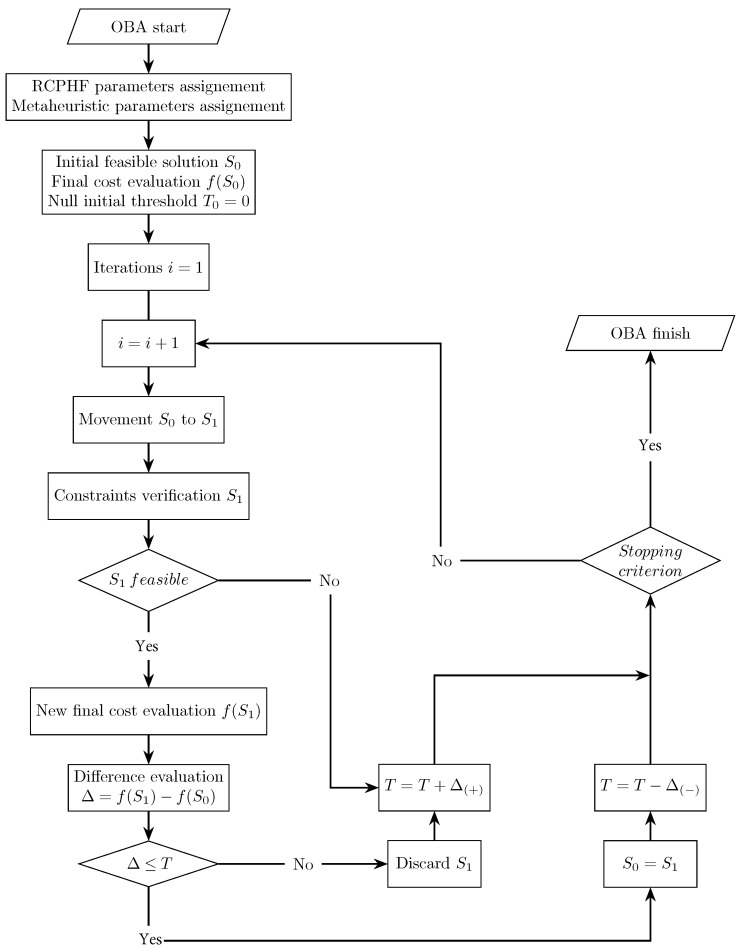
Flowchart of the old bachelor’s acceptance (OBA) process.

The third and last of the metaheuristic techniques applied in the present study is the so-called old bachelor’s acceptance algorithm, hereafter referred to as OBA. The OBA is a modified version of the TA algorithm described in Section 3.2. Instead of starting with a relatively high initial threshold that geometrically decreases over time, Hu et al. [43] proposed a modified algorithm which starts with a null threshold that then decreases or increases according to the acceptance or rejection of new solutions [44]. This quality allows the OBA algorithm to interweave exploration and intensification phases, which can be particularly interesting in problems of specific characteristics. In this context, the operation of the programmed OBA can be seen in Figure 7. The algorithm starts with a randomly generated initial feasible solution $S_{0}$ for which the value of the objective function is evaluated $f(S_{0})$. Then, a new solution is generated by applying a move, and its objective function $f(S_{1})$ is evaluated and compared with the current one. If the new solution improves the current solution or worsens it by a magnitude below the threshold at that time, the new solution is accepted and proceeds to be verified. It is accepted as the current solution if it is a feasible solution. Otherwise, it is rejected. In case the new solution is of better quality than the previous one, the OBA algorithm considers that the current region of the solution space can contain a good quality optimum. Thus, it is interesting to start an intensification phase, which is achieved by reducing the threshold at the rate of a pre-set variation factor $\Delta_{\left(-\right)}$. This factor is applied each time the new solution is better than the previous one. On the other hand, if the new solution worsens the current one, the OBA considers that the current region of the space solution does not present particularly interesting qualities. Therefore, the algorithm must explore in order to find an optimum solution. In order to initiate the exploration phase, the threshold is increased at the variation factor rate $\Delta_{\left(+\right)}$. Unlike the described SA and TA algorithms, the OBA does not have a control parameter that decreases over time. Thus, the stopping criterion limits the process’s duration by setting a maximum number of iterations (M).

## 4. Results

Section 2 provided a comprehensive overview of the combinatorial optimization problem posed in the present study. This includes the complete definition of the parameters, variables, constraints and objective function of the problem. In addition, Section 3 described the operation of the three metaheuristic techniques applied in order to solve it. This section presents the results obtained for the economic optimization of the RCPHF. These are referred to as the final cost of the frame, considered as the objective function of the optimization problem, as well as the associated CO_2_ emissions.

### 4.1. SA Results

The SA algorithm, described in the previous section, starts with an initial feasible solution that it randomly generated. In addition, it requires the definition of the metaheuristic parameters, such as initial temperature, length of Markov’s chain, cooling coefficient and stop or termination criterion. Following the process mentioned in Section 3.1 [40], an arbitrary temperature equivalent to five per cent of the final cost of the initial solution is set. Then, a Markov chain is run at that temperature, and the acceptance rate of worse solutions using the Boltzmann function is evaluated. If the said rate is below a specific lower limit, the algorithm is “cold”. It presents no exploration capability, which is solved by doubling the temperature and repeating the process.

On the other hand, if the acceptance rate exceeds an upper limit, the SA algorithm is too “hot”, which can lead to the loss of interesting solution qualities. To overcome this, the temperature is halved, and another Markov chain is run. This initial temperature-setting process is repeated until the acceptance rate is between 20% and 40%, at which point the initial temperature of the problem is fixed. Nine heuristics are used with Markov chain lengths of 500, 1000 and 5000 iterations and cooling coefficients of 0.80, 0.90 and 0.95. Two stopping criteria are established, the first is to terminate the process when the temperature reaches 5% of the initial temperature. Furthermore, the second is to terminate if, after five consecutive Markov chains, no solution of higher quality than the current one has been generated.

Table 4 presents the costs and associated CO_2_ emissions and average and minimum values, obtained by each of the applied SA algorithms. In addition, the specific parameters of each of the metaheuristics are detailed.

**Figure 8:**
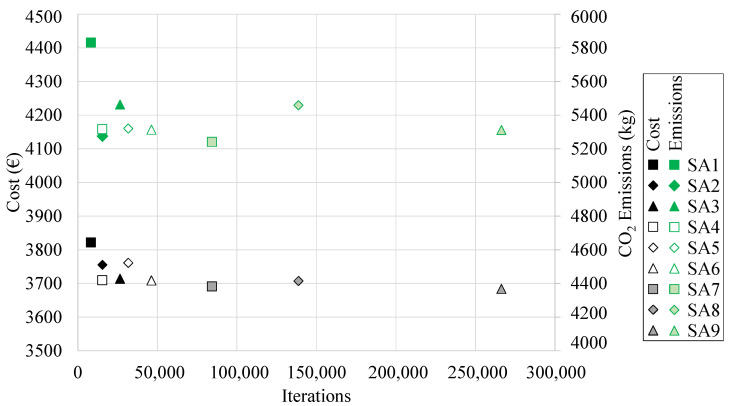
Minimum final cost and associated CO_2_ emissions obtained by the SA algorithm.

The minimum values obtained by each of the nine metaheuristics are represented in Figure 8 as a function of the computation cost. The best result regarding the final cost of the RCPHF was obtained by applying the metaheuristic SA9. With a Markov chain length of 5000 iterations and a cooling coefficient of 0.95, this SA algorithm provides a feasible optimal solution with a final cost of EUR 3863.84 per linear meter. Generally, it can be noted that the SA algorithms with longer chain lengths manage to reach the best quality solutions. However, it is interesting to assess to what extent the computational cost associated with such lengths compensates for reductions in the final cost that may not be sufficiently large.

Concerning the associated emissions, the optimal frame generates roughly 5.3 tons of CO_2_ that are emitted to the atmosphere as a result of its manufacture. With 5.24 tons of CO_2_, the RCPHF design obtained with the SA7 algorithm presents slightly lower emissions. Both this and the aforementioned design use C25/30 concrete and B 500 S steel. The main difference is that the SA7 design presents denser reinforcement while using smaller concrete amounts compared to the SA9 design with the lowest final cost. As stated in Section 1, this is somewhat to be expected since most of the associated CO_2_ emissions originate in cement fabrication.

### 4.2. TA Results

Moving on to the results obtained by applying the second metaheuristic technique, the same procedure as the one described for the SA is considered in the definition of the initial threshold of the TA algorithm. Using the method proposed in [40], an arbitrary threshold equivalent to 0.5% of the final cost of the randomly generated initial feasible solution $S_{0}$ is set. Once the initial threshold is set, the TA runs a chain, and the acceptance rate by threshold comparison is evaluated. If the rate is lower than 20%, the algorithm will not be able to accept solutions that worsen the current one, which can lead to premature convergence to a low-quality local optimum. In order to avoid this, the threshold is doubled and the process restarted. On the other hand, in case the acceptance rate is above 40%, the TA algorithm will accept solutions much worse than the current one.This is something that could mean the loss of interesting qualities of a solution that conforms to a high-quality optimum. To avoid this, the threshold is halved, and a new chain is run. As in the SA algorithm, this process is repeated until the acceptance rate sits between 20% and 40%, the moment at which the algorithm starts the optimization process. Nine different algorithms are developed combining chain lengths of 500, 1000 and 5000 iterations and threshold reduction coefficients of 0.80, 0.90 and 0.95. A stopping criterion is set when the threshold reaches 5% of the initial threshold. A termination criterion is set in case new solutions fail to improve the current one in five consecutive chains.

**Figure 9:**
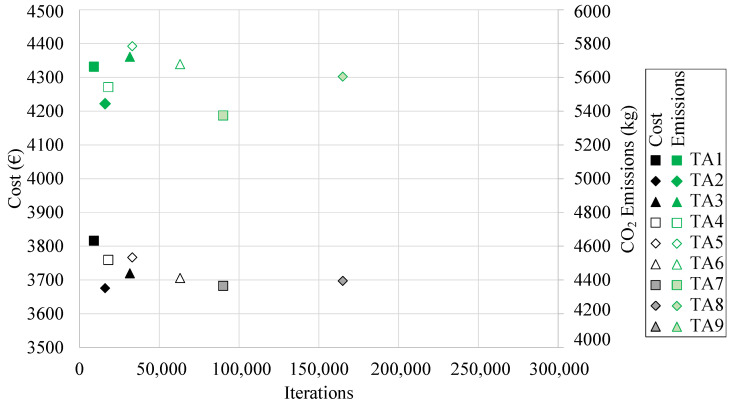
Minimum final cost and associated CO_2_ emissions obtained by the TA algorithm.

Table 4 shows the minimum and average final cost and CO_2_ associated emissions values obtained by applying each of the TA algorithms in the economic optimization of the RCPHF. In addition, each of the heuristic parameters that configure the algorithms is detailed. The minimum cost values as a function of the computation cost are shown in Figure 9.

The RCPHF with the lowest final cost was obtained by employing the TA2 algorithm. This is particularly interesting as said TA algorithm presents a chain length of 500 iterations, locating it in the lower bound of the chain length values. The optimum frame has a final cost of EUR 3675.73 and results in the emission of 5268 kg of CO_2_ into the atmosphere. In this case, the RCPHF with the lowest final cost also happens to be the one that incurs the most negligible CO_2_ emissions. This shows that in most cases minimal cost matches with the lowest CO_2_ associated emission values.

It is of particular relevance to note that with an associated computational cost considerably higher than the TA2 algorithm the TA9 metaheuristic manages to achieve a slightly higher final cost design. This, together with other observations concerning the performance of each of the designed algorithms and the particular characteristics of the optimal RCPHF are detailed in the discussion of the results.

### 4.3. OBA Results

This section focuses on the results obtained through the third and last of the metaheuristic techniques applied in the economic optimization of the RCPHF. As stated in Section 3.3, the OBA algorithm starts with a randomly generated initial feasible solution and an initial threshold equivalent to zero. After this, depending on the acceptance or rejection of new solutions, the threshold varies at the rate of the variation coefficients. In the case of acceptance, the threshold is decreased by $\Delta_{\left(-\right)}$, and in the case of rejection, the threshold is increased by $\Delta_{\left(+\right)}$. The initial implementation of the OBA algorithm considered a single magnitude equivalent to five monetary units for both of the variation coefficients. However, initial runs of the OBA algorithm showed an apparent lack of convergence to good quality optima. An initial study of the process noted that this was a direct consequence of the high exploration capability of the algorithm since the threshold increased excessively when a new solution was rejected. This negatively affected the OBA’s capability to initiate appropriate intensification phases. In order to overcome this lack of convergence, differentiated values for the variation coefficients $\Delta_{\left(-\right)}$ and $\Delta_{\left(+\right)}$ were considered, maintaining the EUR 5 coefficient $\Delta_{\left(-\right)}$ while limiting the $\Delta_{\left(+\right)}$ to one-fifth of its previous value. This modification in the metaheuristic’s parameters manages to sufficiently limit the exploration capability of the OBA algorithm, allowing it to enter prolonged intensification phases and study high-quality solutions.

**Figure 10:**
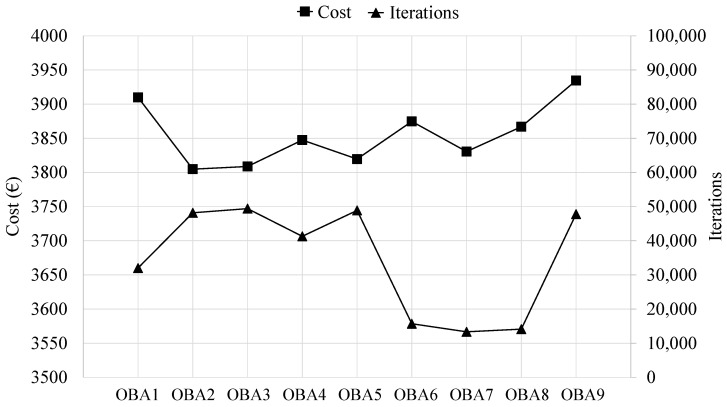
Minimum final cost and associated computational cost for each of the OBA algorithm runs.

Once tuned, considering a termination criterion of 500,000 iterations, the OBA algorithm is applied nine different times, obtaining the results presented in Table 4. These results encompass the minimum and average final cost and associated CO_2_ emission values obtained by applying each of the nine OBA algorithms. In this context, Figure 10 shows the evolution of the final cost obtained by each OBA algorithm as a function of the computational cost associated with obtaining each of these optimal RCPHF designs.

The optimum RCPHF presents a final cost of EUR 3804.83 per linear meter, incurring 5.7 tons of CO_2_ emission into the atmosphere. The optimal design reached by the OBA algorithm presents a slightly higher final cost than those obtained by the SA and TA algorithms. Although the OBA design might indeed be able to improve traditional designs used for the considered structural typology, it is interesting to evaluate which of the metaheuristics applied achieves the best results. This, together with other considerations, is detailed in the following section.

## 5. Discussion

This section discusses the results obtained by applying each of the three metaheuristic techniques described in Section 3 to the combinatorial optimization problem detailed in Section 2. Thus, Table 5 summarises the final costs and associated CO_2_ emissions obtained as a result of the RCPHF economic optimization developed in the present study. In addition, some of the most relevant characteristics of the RCPHF, such as the upper slab and lateral wall depths and the upper slab flexural reinforcement area are presented.

Considering the unit costs shown in Table 1, a reference RCCPF with the same horizontal span, height and earth cover as the RCPHF considered in the present study presents a final cost of EUR 4867.4 per linear meter [30,35]. This means that the algorithms applied in solving the economic optimization achieve reductions of 24.32% in the case of the SA, 24.49% in the TA algorithm and 21.75% by applying the OBA algorithm. In addition, the cast-in-place frame would incur the emission of 7608.97 kg of CO_2_ to the atmosphere, a considerably higher value when compared with the proposed modular structure.

As stated in Section 1, the main fundament behind the economic optimization of the RCPHF is the fact that the final cost is a direct representation of the use of materials. Thus, it also provides a comprehensive representation of the associated CO_2_ emissions. In this context, reducing EUR 1 in the final cost of the RCPHF using the SA algorithm allows for avoiding the emission of 1.94 kg of CO_2_. This corresponds with the avoidance of a 1.96 kg CO_2_ per euro reduction for the TA and 1.72 CO_2_ kg per euro in the case of the OBA algorithm. This is in line with values presented in studies of similar characteristics [20].

Regarding the characteristics of the frames, the RCPHF designs present upper slabs of slightly greater depth when compared to the reference RCCPF. This solves the localized reduction of the flexural reinforcement of the upper slab, an area which is considerably more significant in the traditional cast-in-place design. In the case of the designs obtained by the TA and SA algorithms, the lateral walls are four centimetres slimmer than the cast-in-place reference. However, in the case of the OBA algorithm, the lateral walls present slightly greater depth, being two centimetres greater than the cast-in-place reference frame and a notable six centimetres greater than the TA and SA results.

**Figure 11:**
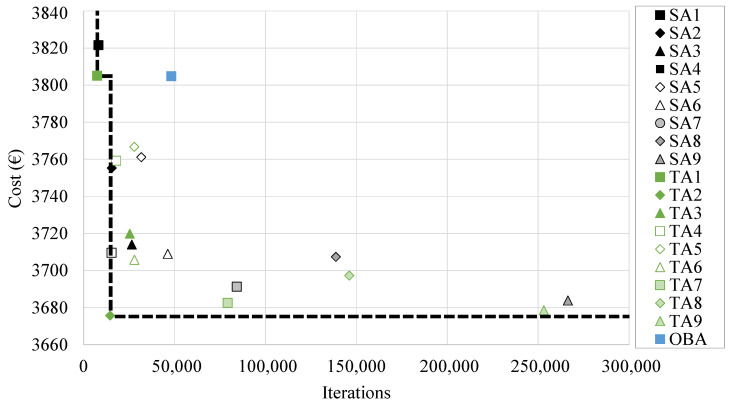
Pareto’s front of the results obtained by the three metaheuristic techniques.

It is particularly relevant to evaluate the performance of the applied metaheuristic techniques. In this context, it is especially interesting to understand that each of the algorithms cannot be evaluated by considering only the final cost of the optimal framework achieved via its application in the resolution of the economic optimization. Thus, given the nature of the metaheuristic techniques, it is especially interesting to evaluate the computational cost associated with obtaining each of these optimal solutions. As stated before, algorithms with longer chains seem to achieve better solutions for the SA algorithm, something similar happening with the TA, even though the minimum cost is achieved using the shorter chain length. However, it is crucial to understand whether the increase in computational cost associated with the higher bounds of such parameters is compensated by the difference in cost compared to other optimal solutions with much lower computational costs. In this context, Figure 11 represents the Pareto front of the combinatorial optimization problem. This corresponds to the union of each of the algorithms that conform to a Pareto optimum when considering the RCPHF final cost and the associated computational cost. In order to be considered so, the algorithm must provide a solution with a restrained final cost while maintaining adequate computational cost. In this context, it can be noted that part of the SA algorithms sit close to the front. However, it is highly conditioned by the TA2 algorithm due to the fact that it obtains a high-quality solution with quite a low computational cost. On the other hand, the OBA algorithm sits considerably far from the area of interest because, considering the computational cost, it does not achieve optimums of sufficient quality that justify its application when compared with the SA or TA algorithms.

The optimal RCPHF obtained by applying the metaheuristic algorithms present several common characteristics. In this context, Table 6 contains the value of each of the different design variables of the optimal frames. It can be noted that the set of optimal RCPHF is characterized by slender sections of reduced depth with a dense passive reinforcement distribution. With reinforcement densities of 91 kg/m^3^ in the case of the SA algorithm result, 89.12 kg/m^3^ for the TA and 86.01 kg/m^3^ in the case of the OBA. Some optimal designs present localized reductions in shear reinforcement, a lack solved by local increases in flexural reinforcement. The passive reinforcement density of the OBA algorithm result is slightly lower than those obtained by applying the rest of the considered metaheuristics. This reduction in the general passive reinforcement of the RCPHF is solved by increasing the upper slab and lateral wall depth. This results in using 6.75% and 5.68% more concrete when compared to the optimal RCPHF obtained by the SA and TA algorithms, respectively.

## 6. Conclusions

In the present study, three metaheuristic techniques were applied to solve the final cost optimization of reinforced concrete hinged precast frames. The study’s main objective is to improve and extend the current knowledge on this structural typology as a substitute for the traditional cast-in-place reinforced concrete frames. This is achieved by evaluating the CO_2_ emissions associated with the optimal designs obtained under restrictive budgets. In this context, the authors consider it appropriate to draw the following conclusions:

- The TA and SA algorithms generate designs with very similar characteristics, achieving the lowest costs and associated CO_2_ emissions. Reductions of EUR 1 in the final cost for a reference cast-in-place frame are equivalent to the avoidance of emitting 1.94, 1.96 and 1.72 kg of CO_2_ in the case of SA, TA and OBA algorithms, respectively;
- Optimizing the final cost of a precast structure conforms to a suitable methodology aiming to reduce the use of materials. Thus, economic optimization allows the reduction of associated CO_2_ emissions. Economic interest drives the industry, making this an especially interesting approach to optimal design;
- Optimal RCPHF designs present thin sections with reduced depths compared to traditional designs. This is solved with particularly dense passive reinforcement designs, which reach up to 91 kg/m^3^, 89.12 kg/m^3^ and 86.01 kg/m^3^ for the SA, TA and OBA algorithms, respectively;
- The optimal design with the lowest final cost obtained by the OBA algorithm presents a slightly lower passive reinforcement density than those obtained by applying the SA and TA. Upper slab and lateral wall sections with greater depths compensate for these reductions. Consequently, the final cost of the frame and the associated CO_2_ emissions are higher than other optimal designs;
- The reduction in the use of materials from restricting the RCPHF final cost results in designs with lower self-weight. This also reduces the associated loads, limiting the structural requirements, which allows optimal use of the concrete and steel;
- The SA and TA algorithms found optimal design solutions where the values of the lateral walls and bottom slab take the minimum bound of the established range which may denote interest in slender designs. This is limited by the constructability conditions imposed. However, the study of this event is an avenue for improvement that will be considered in subsequent studies;
- The prefabrication of the considered structural typology conforms to a way of improvement in the use of natural resources when compared to the cast-in-place alternative. A complete study of the Life Cycle Assessment (LCA) of both structures and a parametric study conforms to a line of research under current development.

## Figures and Tables

**Table 1:** Unit cost and associated CO_2_ emissions values [35].

Unit	Material	Unit Cost (€)	Associated CO_2_ Emissions (kg)
m^3^	C25/30 Concrete	88.86	256.66
m^3^	C30/37 Concrete	97.80	277.72
m^3^	C35/45 Concrete	101.83	278.04
m^3^	C40/50 Concrete	104.83	278.04
kg	B 400 S	1.40	0.70
kg	B 500 S	1.42	0.70

**Table 2:** Main parameters considered in the RCPHF optimization.

**Geometrical parameters**		
Free height (m)	*H*	5
Horizontal span (m)	*L*	10
Hinge height (m)	HH	3
Earth cover (m)	HE	1.50
Lower corner reinforcement length (m)	CR1	2
	CR3	2
Upper corner reinforcement length (m)	CR2	1.5
	CR4	3
Upper slab shear reinforcement length (m)	SR1	3
Lower slab shear reinforcement length (m)	SR2	2
Upper slab flexural reinforcement length (m)	BR1	5
**Loading parameters**		
Earth specific weight (kN/m^3^)	$\gamma_{E}$	20
Reinforced concrete specific weight (kN/m^3^)	$\gamma_{C}$	25
Earth internal friction angle (${}^{\circ }$)	IF	30
Coefficient of active earth pressure	$K_{A}$	0.33
Coefficient of resting earth pressure	$K_{R}$	0.50
Heavy traffic vehicle load (kN/m^3^)	TL	150
Heavy traffic vehicle load length (m)	TLL	1.20
Uniform overload (kN/m^3^)	UO	10
Ballast coefficient (MN/m^3^)	$B_{E}$	10
**Economic and CO_2_ emissions parameters**		
Unit costs (€)	*c_i_*	Table 1
**Exposure-related parameters**		
Exposure class	XC2	
**Legislative-related parameters**		
Standard regulations	CEN [36,37]/MFOM [38]	
Code considerations	MFOM [28]	

**Table 3:** Variables cosidered in the optimization problem.

Geometrical Variables			Num. Values	Range Values
Upper slab depth	(m)	$D_{US}$	41	0.40 to 1.20
Lower slab depth	(m)	$D_{LS}$	41	0.40 to 1.20
Lateral walls depth	(m)	$D_{LW}$	41	0.40 to 1.20
**Materials variables**				
Concrete grade	(MPa)	*C*	4	25 to 40
Steel grade	(MPa)	*S*	2	400 or 500
**Passive reinforcement variables**				
Flexural reinforcement $R_{1}$	(mm)	$\phi_{R_{1}}$	6	10 to 32
	(bars)	$n_{R_{1}}$	9	4 to 12
Flexural reinforcement $R_{2}$	(mm)	$\phi_{R_{2}}$	6	10 to 32
	(bars)	$n_{R_{2}}$	9	4 to 12
Flexural reinforcement $R_{3}$	(mm)	$\phi_{R_{3}}$	6	10 to 32
	(bars)	$n_{R_{3}}$	9	4 to 12
Flexural reinforcement $R_{4}$	(mm)	$\phi_{R_{4}}$	6	10 to 32
	(bars)	$n_{R_{4}}$	9	4 to 12
Flexural reinforcement $R_{5}$	(mm)	$\phi_{R_{5}}$	6	10 to 32
	(bars)	$n_{R_{5}}$	9	4 to 12
Flexural reinforcement $R_{6}$	(mm)	$\phi_{R_{6}}$	6	10 to 32
	(bars)	$n_{R_{6}}$	10	3 to 12
Flexural reinforcement $R_{7}$	(mm)	$\phi_{R_{7}}$	6	10 to 32
	(bars)	$n_{R_{7}}$	9	4 to 12
Flexural reinforcement $R_{8}$	(mm)	$\phi_{R_{8}}$	6	10 to 32
	(bars)	$n_{R_{8}}$	9	4 to 12
Flexural reinforcement $R_{9}$	(mm)	$\phi_{R_{9}}$	6	10 to 32
	(bars)	$n_{R_{9}}$	9	4 to 12
Flexural reinforcement $R_{10}$	(mm)	$\phi_{R_{10}}$	6	10 to 32
	(bars)	$n_{R_{10}}$	10	3 to 12
Flexural reinforcement $R_{11}$	(mm)	$\phi_{R_{11}}$	6	10 to 32
	(bars)	$n_{R_{11}}$	10	3 to 12
Shear reinforcement $R_{12}$	(mm)	$\phi_{R_{12}}$	7	8 to 32
	(m)	$s_{R_{12}}$	7	0.10 to 0.40
Shear reinforcement $R_{13}$	(mm)	$\phi_{R_{13}}$	7	8 to 32
	(m)	$s_{R_{13}}$	7	0.10 to 0.40

**Table 4:** Algorithm parameters in addition to the minimum and mean final cost and associated CO_2_ emissions results obtained.

**SA Algorithm**	**Markov’s Chain**	**Temperature** **Coefficient**	**Iterations**	**Minimum** **Cost (€)**	**Mean Cost (€)**	**Minimum** **CO_2_ (kg)**	**Mean CO_2_ (kg)**
SA1	500	0.80	8158	3821.57	4003.31	5831.25	6200.67
SA2	500	0.90	15,451	3755.27	3828.82	5274.81	5596.65
SA3	500	0.95	26,497	3713.99	3804.87	5463.01	5611.49
SA4	1000	0.80	15,338	3709.59	3837.97	5316.74	5667.25
SA5	1000	0.90	31,686	3761.14	3825.08	5320.67	5660.71
SA 6	1000	0.95	46,246	3708.99	3810.27	5314.10	5725.23
SA7	5000	0.80	84,257	3691.30	3787.28	5241.13	5455.81
SA8	5000	0.90	138,728	3707.36	3769.21	5458.70	5585.77
SA9	5000	0.95	266,363	3683.84	3749.43	5311.64	5569.85
**TA Algorithm**	**Chain**	**Threshold** **coefficient**	**Iterations**	**Minimum** **cost (€)**	**Mean cost (€)**	**Minimum** **CO_2_ (kg)**	**Mean CO_2_ (kg)**
TA1	500	0.80	7416	3805.03	3984.38	5663.23	6080.31
TA2	500	0.90	14,517	3675.73	3873.48	5268.30	5747.97
TA3	500	0.95	25,376	3719.86	3797.42	5366.11	5665.19
TA4	1000	0.80	17,857	3759.21	3854.84	5437.94	5776.78
TA5	1000	0.90	27,859	3766.73	3819.90	5476.89	5732.29
TA6	1000	0.95	27,913	3705.83	3771.47	5290.85	5672.44
TA7	5000	0.80	79,219	3682.57	3741.59	5290.80	5586.77
TA8	5000	0.90	146,111	3697.23	3762.97	5292.91	5648.35
TA9	5000	0.95	253,145	3678.59	3711.77	5372.34	5488.02
**OBA Algorithm**	**Iteration limit**		**Iterations**	**Minimum** **cost (€)**	**Mean cost ()**	**Minimum** **CO_2_ (kg)**	**Mean CO_2_ (kg)**
OBA	500,000		48,195	3804.83	3855.26	5714.00	5637.64
OBA1	500,000		32,028	3909.94	-	5430.45	-
OBA2	500,000		48,195	3804.82	-	5714.00	-
OBA3	500,000		49,409	3808.72	-	5785.66	-
OBA4	500,000		41,295	3847.44	-	5472.21	-
OBA5	500,000		48,878	3819.64	-	5551.71	-
OBA6	500,000		15,698	3874.68	-	6000.80	-
OBA7	500,000		13,336	3830.57	-	5927.97	-
OBA8	500,000		14,118	3866.86	-	5522.67	-
OBA9	500,000		47,809	3934.59	-	5333.26	-

**Table 5:** Summary of the reference and the SA, TA and OBA algorithms results.

	Reference	SA	TA	OBA
Final cost (€)	4867.64	3683.84	3675.73	3804.83
Associated CO_2_ emissions (kg)	7608.97	5311.64	5268.30	5714.00
Upper slab depth (m)	0.75	0.82	0.82	0.86
Lateral walls depth (m)	0.44	0.40	0.40	0.46
Upper slab flexural reinforcement (mm^2^)	4785.50	2827.43	2827.43	2412.74
Final cost reduction (%)	-	24.32	24.49	21.75
Associated CO_2_ emissions reduction (%)	-	30.19	30.76	24.90

**Table 6:** Characteristics of the optimum RCPHF obtained by each metaheuristic technique.

Geometrical Variables			SA	TA	OBA
Upper slab depth	(m)	$D_{US}$	0.82	0.82	0.86
Lower slab depth	(m)	$D_{LS}$	0.40	0.42	0.46
Lateral walls depth	(m)	$D_{LW}$	0.40	0.40	0.46
**Materials variables**					
Concrete grade	(MPa)	*C*	25	25	25
Steel grade	(MPa)	*S*	500	500	500
**Passive reinforcement variables**					
Flexural reinforcement $R_{1}$	(mm)	$\phi_{R_{1}}$	16	12	16
	(bars)	$n_{R_{1}}$	7	12	6
Flexural reinforcement $R_{2}$	(mm)	$\phi_{R_{2}}$	12	10	16
	(bars)	$n_{R_{2}}$	7	10	4
Flexural reinforcement $R_{3}$	(mm)	$\phi_{R_{3}}$	10	10	12
	(bars)	$n_{R_{3}}$	8	8	7
Flexural reinforcement $R_{4}$	(mm)	$\phi_{R_{4}}$	16	16	20
	(bars)	$n_{R_{4}}$	7	7	4
Flexural reinforcement $R_{5}$	(mm)	$\phi_{R_{5}}$	20	20	25
	(bars)	$n_{R_{5}}$	9	9	6
Flexural reinforcement $R_{6}$	(mm)	$\phi_{R_{6}}$	20	20	16
	(bars)	$n_{R_{6}}$	9	9	12
Flexural reinforcement $R_{7}$	(mm)	$\phi_{R_{7}}$	12	16	12
	(bars)	$n_{R_{7}}$	11	6	7
Flexural reinforcement $R_{8}$	(mm)	$\phi_{R_{8}}$	20	20	16
	(bars)	$n_{R_{8}}$	5	5	8
Flexural reinforcement $R_{9}$	(mm)	$\phi_{R_{9}}$	10	10	10
	(bars)	$n_{R_{9}}$	4	4	5
Flexural reinforcement $R_{10}$	(mm)	$\phi_{R_{10}}$	12	12	12
	(bars)	$n_{R_{10}}$	8	8	7
Flexural reinforcement $R_{11}$	(mm)	$\phi_{R_{11}}$	10	10	12
	(bars)	$n_{R_{11}}$	4	4	7
Shear reinforcement $R_{12}$	(mm)	$\phi_{R_{12}}$	32	20	32
	(m)	$s_{R_{12}}$	0.35	0.15	0.40
Shear reinforcement $R_{13}$	(mm)	$\phi_{R_{13}}$	10	10	8
	(m)	$s_{R_{13}}$	0.35	0.35	0.40

## Data Availability

All the data used in the research can be found in the article.

## Abbreviations

The following abbreviations are used in this manuscript:

RCPHF	Reinforced Concrete Precast Hinged Frame
RCCPF	Reinforced Concrete Cast in Place Frame
ULS	Ultimate Limit State
SLS	Service Limit State
LCA	Life Cycle Assessment
SA	Simulated Annealing
TA	Threshold Accepting
OBA	Old Bachelor’s Acceptance

## References

[1] Khan K., Ishfaq M., Amin M.N., Shahzada K., Wahab N., Faraz M.I. (2022). Evaluation of Mechanical and Microstructural Properties and Global Warming Potential of Green Concrete with Wheat Straw Ash and Silica Fume. Materials.

[2] Boesch M.E., Hellweg S. (2010). Identifying improvement potentials in cement production with life cycle assessment. Environ. Sci. Technol..

[3] Martínez-Muñoz D., García J., Martí J.V., Yepes V. (2022). Optimal design of steel–concrete composite bridge based on a transfer function discrete swarm intelligence algorithm. Struct. Multidiscip. Optim..

[4] Petek Gursel A., Masanet E., Horvath A., Stadel A. (2014). Life-cycle inventory analysis of concrete production: A critical review. Cem. Concr. Compos..

[5] Alhorr Y., Eliskandarani E., Elsarrag E. (2014). Approaches to reducing carbon dioxide emissions in the built environment: Low carbon cities. Int. J. Sustain. Built Environ..

[6] Zareei S.A., Ameri F., Bahrami N., Shoaei P., Moosaei H.R., Salemi N. (2019). Performance of sustainable high strength concrete with basic oxygen steel-making (BOS) slag and nano-silica. J. Build. Eng..

[7] Pons J.J., Penadés-Plà V., Yepes V., Martí J.V. (2018). Life cycle assessment of earth-retaining walls: An environmental comparison. J. Clean. Prod..

[8] Chu S. (2019). Effect of paste volume on fresh and hardened properties of concrete. Constr. Build. Mater..

[9] Chu S. (2021). Development of Infilled Cementitious Composites (ICC). Compos. Struct..

[10] Villca A.R., Soriano L., Borrachero M.V., Payá J., Monzó J.M., Tashima M.M. (2022). Hybrid Lime-Pozzolan Geopolymer Systems: Microstructural, Mechanical and Durability Studies. Materials.

[11] Collins F. (2010). Inclusion of carbonation during the life cycle of built and recycled concrete: Influence on their carbon footprint. Int. J. Life Cycle Assess..

[12] Payá J., Soriano L., Font A., Borrachero Rosado M.V., Nande J.A., Monzo Balbuena J.M. (2021). Reuse of Industrial and Agricultural Waste in the Fabrication of Geopolymeric Binders: Mechanical and Microstructural Behavior. Materials.

[13] Borrachero Rosado M.V., Payá J., Brito S., Segura Y.P., Soriano L., Tashima M.M., Monzó J.M. (2022). Reusing Construction and Demolition Waste to Prepare Alkali-Activated Cement. Materials.

[14] Sivakrishna A., Adesina A., Awoyera P., Rajesh Kumar K. (2020). Green concrete: A review of recent developments. Mater. Today Proc..

[15] Paya-Zaforteza I., Yepes V., González-Vidosa F., Hospitaler A. (2010). On the Weibull cost estimation of building frames designed by simulated annealing. Meccanica.

[16] Sarma K.C., Adeli H. (1998). Cost Optimization of Concrete Structures. J. Struct. Eng..

[17] Hare W., Nutini J., Tesfamariam S. (2013). A survey of non-gradient optimization methods in structural engineering. Adv. Eng. Softw..

[18] Afzal M., Liu Y., Cheng J.C., Gan V.J. (2020). Reinforced concrete structural design optimization: A critical review. J. Clean. Prod..

[19] Arandian B., Iraji A., Alaei H., Keawsawasvong S., Nehdi M.L. (2022). White-Tailed Eagle Algorithm for Global Optimization and Low-Cost and Low-CO_2_ Emission Design of Retaining Structures. Sustainability.

[20] Yepes V., Alcalá J., Perea C., González-Vidosa F. (2008). A parametric study of optimum earth-retaining walls by simulated annealing. Eng. Struct..

[21] Penadés-Plà V., García-Segura T., Yepes V. (2019). Accelerated optimization method for low-embodied energy concrete box-girder bridge design. Eng. Struct..

[22] Molina-Moreno F., García-Segura T., Martí J.V., Yepes V. (2017). Optimization of buttressed earth-retaining walls using hybrid harmony search algorithms. Eng. Struct..

[23] Martínez-Muñoz D., Martí J.V., García J., Yepes V. (2021). Embodied Energy Optimization of Buttressed Earth-Retaining Walls with Hybrid Simulated Annealing. Appl. Sci..

[24] Martinez-Martin F.J., Gonzalez-Vidosa F., Hospitaler A., Yepes V. (2012). Multi-objective optimization design of bridge piers with hybrid heuristic algorithms. J. Zhejiang Univ. Sci. A.

[25] Yeo D., Gabbai R.D. (2011). Sustainable design of reinforced concrete structures through embodied energy optimization. Energy Build..

[26] Kaveh A., Izadifard R., Mottaghi L. (2020). Optimal design of planar RC frames considering CO_2_ emissions using ECBO, EVPS and PSO metaheuristic algorithms. J. Build. Eng..

[27] Yepes V., Dasí-Gil M., Martínez-Muñoz D., López-Desfilis V.J., Martí J.V. (2019). Heuristic Techniques for the Design of Steel-Concrete Composite Pedestrian Bridges. Appl. Sci..

[28] MFOM (2009). Guía de Cimentaciones en Obra de Carretera.

[29] Carbonell A., González-Vidosa F., Yepes V. (2011). Design of reinforced concrete road vaults by heuristic optimization. Adv. Eng. Softw..

[30] Perea C., Yepes V., Alcalá J., Hospitaler A., González-Vidosa F. (2010). A parametric study of optimum road frame bridges by threshold acceptance. Indian J. Eng. Mater. Sci..

[31] Zhou Y., Zhan Y., Zhu M., Wang S., Liu J., Ning N. (2022). A Review of the Effects of Raw Material Compositions and Steam Curing Regimes on the Performance and Microstructure of Precast Concrete. Materials.

[32] Šantek Bajto J., Štirmer N., Cerković S., Carević I., Kostanić Jurić K. (2021). Pilot Scale Production of Precast Concrete Elements with Wood Biomass Ash. Materials.

[33] Lu W., Peng W.Q., Zhu L., Gao C., Tang Y.D., Zhou Y.W., Su W., Zeng B. (2022). Experimental and Numerical Study of Static Behavior of Precast Segmental Hollow Bridge Piers. Materials.

[34] Yepes V., Medina J. (2006). Economic heuristic optimization for heterogeneous fleet VRPHESTW. J. Transp. Eng..

[35] BEDEC, Catalonia Institute of Construction Technology, ITeC (2020). BEDEC Materials Database. https://metabase.itec.cat/vide/es/bedec.

[36] (2013). Eurocode 2: Design of Concrete Structures.

[37] (2009). Eurocode 1: Actions on Structures.

[38] MFOM (2011). IAP-11: Code Onthe Actions for the Design of Road Bridges.

[39] Kirkpatrick S., Gelatt C.D., Vecchi M.P. (1983). Optimization by Simulated Annealing. Science.

[40] Medina J. (2001). Estimation of Incident and Reflected Waves Using Simulated Annealing. J. Waterw. Port Coast. Ocean. Eng..

[41] Dueck G., Scheuer T. (1990). Threshold accepting: A general purpose optimization algorithm appearing superior to simulated annealing. J. Comput. Phys..

[42] Yepes V., Gonzalez-Vidosa F., Alcala J., Villalba P. (2012). CO_2_-Optimization Design of Reinforced Concrete Retaining Walls Based on a VNS-Threshold Acceptance Strategy. J. Comput. Civ. Eng..

[43] Hu T.C., Kahng A.B., Tsao C.W.A. (1995). Old Bachelor Acceptance: A New Class of Non-Monotone Threshold Accepting Methods. ORSA J. Comput..

[44] Martínez-Martín F.J., Yepes V., González-Vidosa F., Hospitaler A., Alcalá J. (2022). Optimization Design of RC Elevated Water Tanks under Seismic Loads. Appl. Sci..

